# 1,3-Di-1-adamantylimidazolium (phthalocyaninato)lithium(I) acetone hemisolvate monohydrate

**DOI:** 10.1107/S1600536808041135

**Published:** 2008-12-17

**Authors:** David A Grossie, William A. Feld, John Kelley

**Affiliations:** a Department of Chemistry, Wright State University, 3640 Colonel Glenn Hwy., Dayton Ohio 45435, USA

## Abstract

The asymmetric unit of the title compound, (C_23_H_33_N_2_)[Li(C_32_H_16_N_8_)]·0.5C_3_H_6_O·H_2_O, consists of two symmetry-unrelated lithium phthalocyanine (LiPc^−^) half-anions, centered at (1,0,0) and (0,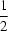,0), respectively, the bis­(adamant­yl)imidazolium cation (BAI^+^), occupying a general site, an acetone mol­ecule, disordered about the inversion centre at (0, 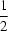, 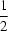) and a water mol­ecule at a general site. The LiPc^−^ anions pack in a stepped pattern enclosing the bis­(adamant­yl)imidazolium cation. Attractions between the anion and cation are mediated by a water mol­ecule which forms O—H⋯N hydrogen bonds.  In addition, two C—H⋯O interactions are seen.

## Related literature

Similar compounds utilizing nitro­gen-based cations have been reported by Homborg & Kalz (1978*a* ▶,*b* ▶). For related structures see: Grossie *et al.* (2006 ▶).
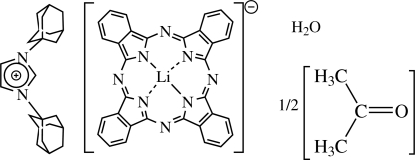


## Experimental

### 

#### Crystal data

(C_23_H_33_N_2_)[Li(C_32_H_16_N_8_)]·0.5C_3_H_6_O·H_2_O
*M* *_r_* = 904.04
Monoclinic, 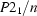

*a* = 15.799 (3) Å
*b* = 17.165 (4) Å
*c* = 17.831 (4) Å
β = 108.374 (3)°
*V* = 4588.9 (16) Å^3^
*Z* = 4
Mo *K*α radiation
μ = 0.08 mm^−1^
*T* = 173 (2) K
0.44 × 0.39 × 0.20 mm

#### Data collection

Bruker Smart APEXII CCD diffractometer
Absorption correction: multi-scan (*SADABS*; Bruker, 2003 ▶) *T*_min_ = 0.901, *T*_max_ = 0.985
54676 measured reflections
14917 independent reflections
11038 reflections with *I* > 2σ(*I*)
*R*_int_ = 0.039

#### Refinement

*R*[*F* ^2^ > 2σ(*F* ^2^)] = 0.052
*wR*(*F* ^2^) = 0.129
*S* = 1.03
14917 reflections
649 parameters
8 restraints
H atoms treated by a mixture of independent and constrained refinement
Δρ_max_ = 0.37 e Å^−3^
Δρ_min_ = −0.30 e Å^−3^

### 

Data collection: *SMART* (Bruker, 2002 ▶); cell refinement: *SAINT-Plus* (Bruker, 2003 ▶); data reduction: *SAINT-Plus*; program(s) used to solve structure: *SHELXS97* (Sheldrick, 2008 ▶); program(s) used to refine structure: *SHELXL97* (Sheldrick, 2008 ▶); molecular graphics: *Mercury* (Macrae *et al.*, 2006 ▶), *ORTEP-3 for Windows*, (Farrugia, 1997 ▶), *OSCAIL*, (McArdle, 1995 ▶); software used to prepare material for publication: *enCIFer* (Allen *et al.*, 2004 ▶) and *publCIF* (Westrip, 2009 ▶).

## Supplementary Material

Crystal structure: contains datablocks I, New_Global_Publ_Block. DOI: 10.1107/S1600536808041135/sj2558sup1.cif

Structure factors: contains datablocks I. DOI: 10.1107/S1600536808041135/sj2558Isup2.hkl

Additional supplementary materials:  crystallographic information; 3D view; checkCIF report

## Figures and Tables

**Table 1:** Hydrogen-bond geometry (Å, °)

*D*—H⋯*A*	*D*—H	H⋯*A*	*D*⋯*A*	*D*—H⋯*A*
O1—H1*A*⋯N6	0.861 (18)	2.405 (17)	3.1618 (17)	147.1 (18)
O1—H1*B*⋯N3^i^	0.889 (18)	2.025 (19)	2.8911 (17)	164.3 (16)
C33—H33⋯O1	0.95	2.18	3.127 (2)	171
C37—H37*A*⋯O1	0.99	2.52	3.493 (2)	166

Symmetry code: (i) 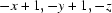.

**Fig. 1.**
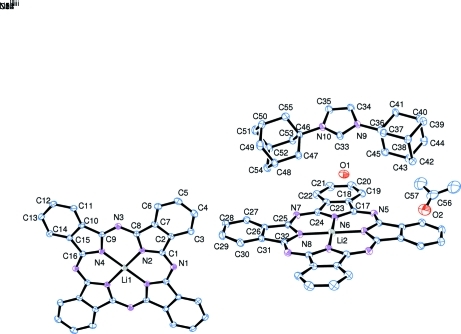
The asymmetric unit of I. Labelled atoms are related to unlabelled atoms by the symmetry codes –x+2, -y, -z for the Li1 anion and -x, -y+1, -z for the Li2 anion. Hydrogen atoms are not shown.

**Fig. 2.**
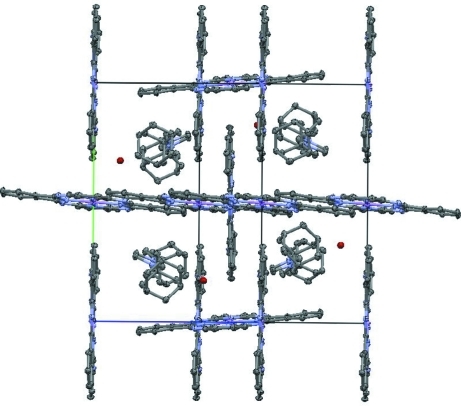
Unit cell packing diagram, viewed along the b axis. Acetone molecules have been omitted for clarity.

## supplementary crystallographic information

### Comment

The asymmetric unit of the title compound consists of two symmetry-unrelated halves of lithium phthalocyanine anions (LiPc^-^), centered at 1,0,0 and 0, 1/2,0, respectively; the bis(adamantyl)imidazolium cation (BAI^+^), occupying a general site, an acetone molecule, at 0, 1/2,0.5, and a water molecule at a general site, Fig 1. In addition, the unit cell packing provides interesting detail of the arrangement of molecules within the crystal structure, as seen in Figure 2. Similar compounds utilizing nitrogen-based cations have been reported by Homborg & Kalz (1978a, 1978b).

Although they may appear to be in Figure 2, the symmetry-related LiPc^-^ anions are not parallel, as seen in the angle (2.39°) between their mean planes. Additionally, their intermolecular distances are quite large (10.10 Å as measured between mesonitrogens) in comparison to those seen in Li_2_PC (3.06–3.38 Å)(Grossie, *et al.*, 2006) The large spacing between LiPc^-^ molecules is easily attributed to the bulky adamantyl substituents of the BAI^+^ molecules, in which one cation appears to be enclosed within four LiPc^-^ anions forming ionic pockets. This is shown in Fig. 2, which presents the organized but unique packing of molecules. Rows of symmetry-related anions along the *b* axis are offset from each other, stacking in a stair-step manner. These molecules appear to be nearly orthogonal to the columnar anions, in which alternating columns have slightly different orientations. It is necessary, though, to view the packing from all angles to get a true understanding of ion arrangements.

Probably the most intriguing information obtained was the role of solvent molecules within the crystal structure. It was seen in the crystal structure of Li_2_PC that acetone and water ligated to lithium, forming dimers that were found in between LiPc^-^ pairs. In the current structure it can be seen that water molecules are crucial to the crystallization of the complex ions. Here, it is noticed that water forms O—H···N and C—H···O hydrogen bonds, Table 1, with the two symmetry-unrelated LiPc^-^ anions and one BAI^+^ cation, acting as an intermediate to the three ions. Interatomic distances between hydrogen atoms and isoindoline and *meso* nitrogen of individual LiPc^-^ ions were found to be 2.405 (17) Å and 2.025 (19) Å, respectively. The distance between the oxygen atom of water and the hydrogen on the 2- position of the BAI^+^ cation was calculated to be about 2.18 Å.

### Experimental

The 1,3-bis(1-adamantyl)imidazolium tetrafluoroborate (0.884 g) was purified by dissolving it in 70 ml of acetone and filtering the insoluble impurities. The solution was evaporated to dryness to give 0.843 g (1.98 mmol) of the pure salt, which was redissolved in 10 ml of acetone and added to a solution of 0.991 g (1.98 mmol) of dilithium phthalocyanine in 100 ml of acetone. The solution was evaporated to approximately 20–30 ml under reduced pressure to the point of crystallization, sealed and crystallized at 5°C for 72 h. The resulting solid was redissolved in 125 ml of hot acetone with stirring (some undissolved solid remained). The volume was reduced and crystallized at 5°C. The dry product isolated by filtration gave 0.893 g (55.3%) of purple crystals. m.p. 349–351°C. Anal. Calc. for C55H49LiN10 (856.99): C, 77.08; H, 5.76; N, 16.34. Found: C, 76.89; H, 5.90; N, 15.94.

### Refinement

Hydrogen atoms of the water molecule and of the methyl group of the acetone molecule were located in a difference Fourier map and refined with appropriate distance restraints.  All other H-atoms were positioned geometrically and refined using a riding model with d(C-H) = 0.95Å,  U_iso_=1.2U_eq_ (C) for aromatic 1.00Å,  U_iso_ = 1.2U_eq_ (C) for CH and 0.99Å,  U_iso_ = 1.2U_eq_ (C) for CH_2_ atoms

### Crystal data

**Table:**

(C_23_H_33_N_2_)[Li(C_32_H_16_N_8_)]·0.5C_3_H_6_O·H_2_O	*F*(000) = 1912
*M**_r_* = 904.04	*D*_x_ = 1.309 Mg m^−^^3^
Monoclinic, *P*2_1_/*n*	Melting point: 622 K
Hall symbol: -P 2yn	Mo *K*α radiation, λ = 0.71073 Å
*a* = 15.799 (3) Å	Cell parameters from 64888 reflections
*b* = 17.165 (4) Å	θ = 2.4–30.1°
*c* = 17.831 (4) Å	µ = 0.08 mm^−^^1^
β = 108.374 (3)°	*T* = 173 K
*V* = 4588.9 (16) Å^3^	Block, violet
*Z* = 4	0.44 × 0.39 × 0.20 mm

### Data collection

**Table:**

Bruker Smart APEXII CCD diffractometer	14917 independent reflections
Radiation source: fine-focus sealed tube	11038 reflections with *I* > 2σ(*I*)
graphite	*R*_int_ = 0.039
ω scans	θ_max_ = 31.7°, θ_min_ = 1.7°
Absorption correction: multi-scan (*SADABS*; Bruker, 2003)	*h* = −22→23
*T*_min_ = 0.901, *T*_max_ = 0.985	*k* = −25→24
54676 measured reflections	*l* = −26→26

### Refinement

**Table:**

Refinement on *F*^2^	Primary atom site location: structure-invariant direct methods
Least-squares matrix: full	Secondary atom site location: difference Fourier map
*R*[*F*^2^ > 2σ(*F*^2^)] = 0.052	Hydrogen site location: inferred from neighbouring sites
*wR*(*F*^2^) = 0.129	H atoms treated by a mixture of independent and constrained refinement
*S* = 1.03	*w* = 1/[σ^2^(*F*_o_^2^) + (0.0482*P*)^2^ + 2.081*P*] where *P* = (*F*_o_^2^ + 2*F*_c_^2^)/3
14917 reflections	(Δ/σ)_max_ = 0.001
649 parameters	Δρ_max_ = 0.37 e Å^−^^3^
8 restraints	Δρ_min_ = −0.29 e Å^−^^3^

### Special details

**Table:**

Experimental. 1H NMR (300 MHz, DMSO-d6) δ = 9.35–9.25 (m, 8H, Ar—H), 9.06 (s, 1H, Ar—H), 8.12–8.02 (m, 8H, Ar—H), 8.01 (d, 2H, 3 J = 1.1 Hz, Ar—H), 2.24–2.09 (m, 18H, Al—H), 1.82–1.62 (m, 12H, Al—H); 13 C-NMR (75 MHz, DMSO-d6) δ = 154.14, 140.05, 131.23, 127.51, 119.31, 59.35, 41.46, 34.81, 28.83; IR (KBr) cm^-1^ = 3053 (Ar—H), 2912 (C—H), 1604 (C—C), 1583 (C—N), 1485 (C—N), 1092 (C—C), 1055 (C—N); UV/Vis (DMSO) λmax nm (log ε) = 665 (5.25), 636 (4.47), 601 (4.48), 380 (4.55), 327 (4.55), 255 (4.63);
Geometry. All e.s.d.'s (except the e.s.d. in the dihedral angle between two l.s. planes) are estimated using the full covariance matrix. The cell e.s.d.'s are taken into account individually in the estimation of e.s.d.'s in distances, angles and torsion angles; correlations between e.s.d.'s in cell parameters are only used when they are defined by crystal symmetry. An approximate (isotropic) treatment of cell e.s.d.'s is used for estimating e.s.d.'s involving l.s. planes.
Refinement. Refinement of *F*^2^ against ALL reflections. The weighted *R*-factor *wR* and goodness of fit *S* are based on *F*^2^, conventional *R*-factors *R* are based on *F*, with *F* set to zero for negative *F*^2^. The threshold expression of *F*^2^ > σ(*F*^2^) is used only for calculating *R*-factors(gt) *etc*. and is not relevant to the choice of reflections for refinement. *R*-factors based on *F*^2^ are statistically about twice as large as those based on *F*, and *R*- factors based on ALL data will be even larger.

### Fractional atomic coordinates and isotropic or equivalent isotropic displacement parameters (Å^2^)

**Table:**

	*x*	*y*	*z*	*U*_iso_*/*U*_eq_	Occ. (<1)
Li1	1.0000	0.0000	0.0000	0.0221 (7)	
N1	0.84511 (7)	0.01780 (6)	0.09200 (6)	0.0183 (2)	
N2	0.94090 (7)	0.08811 (6)	0.03257 (6)	0.0173 (2)	
N3	1.01005 (7)	0.19613 (6)	−0.01323 (6)	0.0182 (2)	
N4	1.06695 (7)	0.07380 (6)	−0.04398 (6)	0.0178 (2)	
C1	0.87993 (8)	0.08236 (7)	0.07194 (7)	0.0167 (2)	
C2	0.85314 (8)	0.15984 (7)	0.08970 (7)	0.0173 (2)	
C3	0.79734 (9)	0.18458 (8)	0.13159 (8)	0.0213 (3)	
H3	0.7654	0.1483	0.1526	0.026*	
C4	0.78994 (10)	0.26408 (8)	0.14163 (8)	0.0240 (3)	
H4	0.7524	0.2828	0.1701	0.029*	
C5	0.83698 (10)	0.31696 (8)	0.11036 (9)	0.0256 (3)	
H5	0.8310	0.3711	0.1183	0.031*	
C6	0.89232 (9)	0.29251 (8)	0.06800 (8)	0.0222 (3)	
H6	0.9237	0.3290	0.0466	0.027*	
C7	0.90028 (9)	0.21275 (7)	0.05796 (7)	0.0179 (2)	
C8	0.95462 (8)	0.16503 (7)	0.02226 (7)	0.0173 (2)	
C9	1.06334 (8)	0.15280 (7)	−0.04204 (7)	0.0172 (2)	
C10	1.12886 (8)	0.18684 (7)	−0.07575 (7)	0.0174 (2)	
C11	1.15259 (9)	0.26268 (8)	−0.08836 (8)	0.0201 (2)	
H11	1.1230	0.3062	−0.0750	0.024*	
C12	1.22089 (9)	0.27270 (8)	−0.12104 (8)	0.0221 (3)	
H12	1.2382	0.3240	−0.1301	0.026*	
C13	1.26472 (9)	0.20899 (8)	−0.14097 (8)	0.0228 (3)	
H13	1.3115	0.2177	−0.1629	0.027*	
C14	1.24094 (9)	0.13324 (8)	−0.12920 (8)	0.0213 (3)	
H14	1.2704	0.0898	−0.1428	0.026*	
C15	1.17233 (9)	0.12325 (7)	−0.09671 (8)	0.0181 (2)	
C16	1.13064 (9)	0.05364 (7)	−0.07705 (7)	0.0177 (2)	
Li2	0.0000	0.5000	0.0000	0.0223 (7)	
N5	−0.00519 (7)	0.50482 (6)	0.18826 (6)	0.0193 (2)	
N6	0.08896 (7)	0.49153 (7)	0.10472 (6)	0.0192 (2)	
N7	0.22215 (7)	0.47223 (6)	0.06714 (7)	0.0195 (2)	
N8	0.09266 (7)	0.48310 (7)	−0.04998 (6)	0.0187 (2)	
C17	0.07282 (9)	0.49409 (7)	0.17554 (7)	0.0183 (2)	
C18	0.15585 (9)	0.48247 (7)	0.24040 (8)	0.0187 (2)	
C19	0.17516 (10)	0.47967 (8)	0.32180 (8)	0.0229 (3)	
H19	0.1297	0.4863	0.3457	0.027*	
C20	0.26314 (10)	0.46682 (9)	0.36727 (8)	0.0279 (3)	
H20	0.2782	0.4649	0.4232	0.033*	
C21	0.32991 (10)	0.45674 (9)	0.33197 (9)	0.0286 (3)	
H21	0.3895	0.4475	0.3643	0.034*	
C22	0.31062 (9)	0.45991 (8)	0.25046 (8)	0.0237 (3)	
H22	0.3561	0.4534	0.2266	0.028*	
C23	0.22251 (9)	0.47299 (8)	0.20492 (8)	0.0196 (2)	
C24	0.17785 (9)	0.47881 (7)	0.11946 (8)	0.0187 (2)	
C25	0.18188 (9)	0.47596 (7)	−0.01094 (8)	0.0183 (2)	
C26	0.23144 (9)	0.47344 (7)	−0.06777 (8)	0.0183 (2)	
C27	0.32210 (9)	0.47045 (8)	−0.05927 (8)	0.0213 (3)	
H27	0.3657	0.4665	−0.0086	0.026*	
C28	0.34626 (10)	0.47343 (8)	−0.12769 (9)	0.0251 (3)	
H28	0.4077	0.4719	−0.1235	0.030*	
C29	0.28251 (10)	0.47858 (9)	−0.20269 (9)	0.0255 (3)	
H29	0.3013	0.4799	−0.2483	0.031*	
C30	0.19217 (10)	0.48184 (8)	−0.21123 (8)	0.0224 (3)	
H30	0.1487	0.4857	−0.2619	0.027*	
C31	0.16757 (9)	0.47920 (7)	−0.14276 (8)	0.0185 (2)	
C32	0.08121 (9)	0.48562 (7)	−0.12921 (8)	0.0179 (2)	
N9	0.21347 (8)	0.75837 (7)	0.32337 (7)	0.0206 (2)	
N10	0.31358 (8)	0.73900 (7)	0.26556 (7)	0.0210 (2)	
C33	0.22743 (9)	0.73102 (8)	0.25812 (8)	0.0212 (3)	
H33	0.1832	0.7094	0.2137	0.025*	
C34	0.29366 (9)	0.78481 (9)	0.37394 (8)	0.0238 (3)	
H34	0.3033	0.8071	0.4247	0.029*	
C35	0.35583 (9)	0.77306 (9)	0.33786 (8)	0.0245 (3)	
H35	0.4173	0.7859	0.3584	0.029*	
C36	0.12688 (9)	0.75260 (8)	0.33990 (8)	0.0191 (2)	
C37	0.05180 (9)	0.78827 (8)	0.27195 (8)	0.0205 (3)	
H37A	0.0471	0.7607	0.2220	0.025*	
H37B	0.0649	0.8438	0.2654	0.025*	
C38	−0.03623 (9)	0.78110 (8)	0.29063 (8)	0.0222 (3)	
H38	−0.0855	0.8046	0.2466	0.027*	
C39	−0.02848 (10)	0.82412 (9)	0.36785 (9)	0.0258 (3)	
H39A	−0.0155	0.8799	0.3624	0.031*	
H39B	−0.0856	0.8204	0.3796	0.031*	
C40	0.04642 (10)	0.78776 (9)	0.43521 (8)	0.0262 (3)	
H40	0.0512	0.8156	0.4856	0.031*	
C41	0.13491 (9)	0.79498 (9)	0.41737 (8)	0.0245 (3)	
H41A	0.1491	0.8506	0.4127	0.029*	
H41B	0.1837	0.7717	0.4610	0.029*	
C42	−0.05610 (10)	0.69499 (9)	0.29889 (9)	0.0252 (3)	
H42A	−0.0612	0.6673	0.2489	0.030*	
H42B	−0.1135	0.6896	0.3098	0.030*	
C43	0.01881 (10)	0.65890 (8)	0.36645 (9)	0.0263 (3)	
H43	0.0055	0.6026	0.3719	0.032*	
C44	0.02643 (11)	0.70154 (10)	0.44379 (9)	0.0300 (3)	
H44A	−0.0301	0.6963	0.4563	0.036*	
H44B	0.0748	0.6781	0.4876	0.036*	
C45	0.10709 (10)	0.66639 (8)	0.34806 (9)	0.0242 (3)	
H45A	0.1560	0.6426	0.3912	0.029*	
H45B	0.1027	0.6386	0.2983	0.029*	
C46	0.35926 (9)	0.71388 (8)	0.20826 (7)	0.0188 (2)	
C47	0.29214 (9)	0.67521 (8)	0.13680 (8)	0.0218 (3)	
H47A	0.2441	0.7124	0.1105	0.026*	
H47B	0.2648	0.6295	0.1540	0.026*	
C48	0.34101 (9)	0.64948 (9)	0.07899 (8)	0.0240 (3)	
H48	0.2977	0.6241	0.0319	0.029*	
C49	0.38222 (11)	0.72049 (10)	0.05245 (9)	0.0301 (3)	
H49A	0.3349	0.7583	0.0262	0.036*	
H49B	0.4125	0.7044	0.0140	0.036*	
C50	0.44974 (11)	0.75862 (9)	0.12455 (9)	0.0294 (3)	
H50	0.4773	0.8048	0.1071	0.035*	
C51	0.52255 (10)	0.70054 (10)	0.16613 (9)	0.0302 (3)	
H51A	0.5655	0.7255	0.2128	0.036*	
H51B	0.5553	0.6842	0.1297	0.036*	
C52	0.48106 (9)	0.62939 (9)	0.19228 (8)	0.0243 (3)	
H52	0.5289	0.5912	0.2189	0.029*	
C53	0.43204 (9)	0.65519 (8)	0.24972 (8)	0.0221 (3)	
H53A	0.4747	0.6795	0.2970	0.027*	
H53B	0.4049	0.6094	0.2671	0.027*	
C54	0.41484 (9)	0.59165 (9)	0.11957 (9)	0.0254 (3)	
H54A	0.4463	0.5752	0.0822	0.030*	
H54B	0.3884	0.5448	0.1357	0.030*	
C55	0.40064 (10)	0.78504 (8)	0.18204 (9)	0.0266 (3)	
H55A	0.4430	0.8107	0.2286	0.032*	
H55B	0.3534	0.8230	0.1557	0.032*	
O1	0.06786 (8)	0.67405 (6)	0.11486 (7)	0.0297 (2)	
H1A	0.0548 (13)	0.6284 (10)	0.0945 (12)	0.045*	
H1B	0.0393 (13)	0.7070 (11)	0.0768 (10)	0.045*	
O2	−0.0403 (2)	0.44760 (18)	0.39840 (18)	0.0555 (7)	0.50
C56	−0.0162 (3)	0.4789 (2)	0.4612 (2)	0.0451 (9)	0.50
C57	0.0801 (2)	0.47921 (16)	0.50835 (16)	0.0646 (7)	
H57A	0.1107 (14)	0.4355 (14)	0.4973 (17)	0.097*	
H57B	0.0942 (15)	0.4617 (18)	0.5603 (12)	0.097*	
H57C	0.1131 (14)	0.5205 (14)	0.4976 (16)	0.097*	

### Atomic displacement parameters (Å^2^)

**Table:**

	*U*^11^	*U*^22^	*U*^33^	*U*^12^	*U*^13^	*U*^23^
Li1	0.0250 (17)	0.0152 (14)	0.0268 (17)	−0.0003 (12)	0.0094 (14)	0.0006 (12)
N1	0.0193 (5)	0.0163 (5)	0.0190 (5)	−0.0001 (4)	0.0058 (4)	0.0004 (4)
N2	0.0191 (5)	0.0153 (5)	0.0171 (5)	−0.0011 (4)	0.0052 (4)	0.0003 (4)
N3	0.0199 (5)	0.0168 (5)	0.0177 (5)	0.0000 (4)	0.0056 (4)	0.0007 (4)
N4	0.0191 (5)	0.0151 (5)	0.0194 (5)	−0.0001 (4)	0.0064 (4)	0.0010 (4)
C1	0.0175 (6)	0.0162 (5)	0.0154 (5)	0.0004 (4)	0.0038 (4)	−0.0011 (4)
C2	0.0184 (6)	0.0161 (5)	0.0162 (6)	0.0010 (4)	0.0036 (5)	−0.0009 (4)
C3	0.0215 (6)	0.0214 (6)	0.0214 (6)	0.0017 (5)	0.0073 (5)	0.0006 (5)
C4	0.0251 (7)	0.0235 (6)	0.0239 (7)	0.0061 (5)	0.0085 (5)	−0.0022 (5)
C5	0.0304 (7)	0.0180 (6)	0.0272 (7)	0.0051 (5)	0.0075 (6)	−0.0026 (5)
C6	0.0248 (7)	0.0162 (6)	0.0245 (7)	0.0011 (5)	0.0060 (5)	−0.0001 (5)
C7	0.0191 (6)	0.0163 (5)	0.0168 (6)	0.0019 (4)	0.0035 (5)	−0.0001 (4)
C8	0.0182 (6)	0.0156 (5)	0.0165 (6)	0.0004 (4)	0.0033 (5)	−0.0001 (4)
C9	0.0183 (6)	0.0152 (5)	0.0166 (6)	−0.0016 (4)	0.0034 (5)	0.0001 (4)
C10	0.0185 (6)	0.0168 (5)	0.0156 (5)	−0.0022 (4)	0.0039 (4)	0.0005 (4)
C11	0.0231 (6)	0.0170 (6)	0.0189 (6)	−0.0031 (5)	0.0049 (5)	−0.0008 (5)
C12	0.0242 (7)	0.0197 (6)	0.0213 (6)	−0.0064 (5)	0.0056 (5)	0.0008 (5)
C13	0.0215 (6)	0.0247 (6)	0.0231 (6)	−0.0061 (5)	0.0083 (5)	0.0003 (5)
C14	0.0210 (6)	0.0210 (6)	0.0225 (6)	−0.0013 (5)	0.0075 (5)	−0.0006 (5)
C15	0.0192 (6)	0.0172 (5)	0.0175 (6)	−0.0016 (5)	0.0054 (5)	0.0010 (4)
C16	0.0187 (6)	0.0165 (5)	0.0171 (6)	−0.0011 (4)	0.0046 (5)	0.0001 (4)
Li2	0.0212 (16)	0.0281 (17)	0.0162 (15)	0.0012 (13)	0.0040 (12)	−0.0005 (13)
N5	0.0207 (5)	0.0195 (5)	0.0172 (5)	0.0008 (4)	0.0052 (4)	0.0001 (4)
N6	0.0185 (5)	0.0233 (5)	0.0155 (5)	0.0016 (4)	0.0047 (4)	−0.0006 (4)
N7	0.0193 (5)	0.0208 (5)	0.0176 (5)	0.0020 (4)	0.0046 (4)	−0.0005 (4)
N8	0.0179 (5)	0.0215 (5)	0.0160 (5)	0.0010 (4)	0.0043 (4)	0.0004 (4)
C17	0.0192 (6)	0.0184 (6)	0.0164 (6)	0.0002 (5)	0.0042 (5)	−0.0002 (5)
C18	0.0202 (6)	0.0168 (5)	0.0175 (6)	0.0008 (5)	0.0036 (5)	0.0003 (5)
C19	0.0276 (7)	0.0225 (6)	0.0169 (6)	0.0025 (5)	0.0047 (5)	−0.0004 (5)
C20	0.0320 (8)	0.0305 (7)	0.0163 (6)	0.0040 (6)	0.0004 (6)	0.0013 (5)
C21	0.0241 (7)	0.0334 (8)	0.0218 (7)	0.0057 (6)	−0.0022 (5)	0.0019 (6)
C22	0.0205 (6)	0.0263 (7)	0.0217 (7)	0.0042 (5)	0.0028 (5)	0.0007 (5)
C23	0.0213 (6)	0.0183 (6)	0.0171 (6)	0.0021 (5)	0.0027 (5)	−0.0007 (5)
C24	0.0184 (6)	0.0190 (6)	0.0174 (6)	0.0009 (5)	0.0038 (5)	−0.0001 (5)
C25	0.0180 (6)	0.0181 (6)	0.0185 (6)	0.0016 (5)	0.0052 (5)	−0.0010 (5)
C26	0.0204 (6)	0.0155 (5)	0.0194 (6)	0.0007 (5)	0.0068 (5)	−0.0015 (4)
C27	0.0199 (6)	0.0213 (6)	0.0224 (6)	0.0007 (5)	0.0063 (5)	−0.0025 (5)
C28	0.0218 (7)	0.0266 (7)	0.0296 (7)	0.0014 (5)	0.0122 (6)	−0.0024 (6)
C29	0.0284 (7)	0.0276 (7)	0.0248 (7)	0.0010 (6)	0.0143 (6)	−0.0002 (5)
C30	0.0262 (7)	0.0221 (6)	0.0201 (6)	0.0012 (5)	0.0089 (5)	−0.0007 (5)
C31	0.0205 (6)	0.0154 (5)	0.0203 (6)	0.0012 (5)	0.0074 (5)	−0.0010 (5)
C32	0.0198 (6)	0.0169 (5)	0.0175 (6)	0.0002 (5)	0.0065 (5)	−0.0010 (4)
N9	0.0190 (5)	0.0252 (6)	0.0181 (5)	0.0013 (4)	0.0064 (4)	−0.0031 (4)
N10	0.0189 (5)	0.0261 (6)	0.0180 (5)	0.0018 (4)	0.0059 (4)	−0.0028 (4)
C33	0.0185 (6)	0.0275 (7)	0.0173 (6)	0.0018 (5)	0.0054 (5)	−0.0022 (5)
C34	0.0203 (6)	0.0303 (7)	0.0196 (6)	0.0003 (5)	0.0048 (5)	−0.0059 (5)
C35	0.0198 (6)	0.0318 (7)	0.0206 (6)	−0.0002 (5)	0.0047 (5)	−0.0063 (5)
C36	0.0190 (6)	0.0223 (6)	0.0175 (6)	0.0013 (5)	0.0078 (5)	−0.0012 (5)
C37	0.0211 (6)	0.0226 (6)	0.0187 (6)	−0.0001 (5)	0.0073 (5)	0.0011 (5)
C38	0.0200 (6)	0.0246 (6)	0.0223 (6)	0.0011 (5)	0.0071 (5)	0.0009 (5)
C39	0.0246 (7)	0.0260 (7)	0.0305 (7)	0.0011 (5)	0.0140 (6)	−0.0044 (6)
C40	0.0267 (7)	0.0349 (8)	0.0202 (6)	−0.0023 (6)	0.0120 (5)	−0.0080 (6)
C41	0.0225 (7)	0.0336 (7)	0.0189 (6)	−0.0012 (6)	0.0085 (5)	−0.0069 (5)
C42	0.0247 (7)	0.0276 (7)	0.0256 (7)	−0.0065 (5)	0.0113 (6)	−0.0046 (5)
C43	0.0315 (8)	0.0223 (6)	0.0286 (7)	−0.0010 (6)	0.0146 (6)	0.0030 (5)
C44	0.0327 (8)	0.0385 (8)	0.0226 (7)	0.0008 (6)	0.0142 (6)	0.0050 (6)
C45	0.0283 (7)	0.0219 (6)	0.0243 (7)	0.0036 (5)	0.0108 (6)	0.0020 (5)
C46	0.0176 (6)	0.0239 (6)	0.0161 (6)	0.0005 (5)	0.0072 (5)	−0.0011 (5)
C47	0.0164 (6)	0.0301 (7)	0.0189 (6)	0.0002 (5)	0.0057 (5)	−0.0037 (5)
C48	0.0199 (6)	0.0342 (7)	0.0177 (6)	−0.0007 (5)	0.0057 (5)	−0.0044 (5)
C49	0.0334 (8)	0.0388 (8)	0.0206 (7)	0.0006 (6)	0.0121 (6)	0.0029 (6)
C50	0.0348 (8)	0.0307 (7)	0.0284 (7)	−0.0084 (6)	0.0180 (6)	−0.0012 (6)
C51	0.0200 (7)	0.0452 (9)	0.0278 (7)	−0.0074 (6)	0.0112 (6)	−0.0105 (7)
C52	0.0182 (6)	0.0320 (7)	0.0226 (7)	0.0049 (5)	0.0061 (5)	−0.0035 (5)
C53	0.0194 (6)	0.0274 (7)	0.0194 (6)	0.0027 (5)	0.0058 (5)	−0.0003 (5)
C54	0.0227 (7)	0.0293 (7)	0.0254 (7)	0.0001 (5)	0.0092 (5)	−0.0068 (6)
C55	0.0313 (8)	0.0233 (7)	0.0271 (7)	−0.0022 (6)	0.0121 (6)	−0.0006 (5)
O1	0.0350 (6)	0.0229 (5)	0.0269 (6)	0.0015 (4)	0.0035 (5)	0.0014 (4)
O2	0.071 (2)	0.0482 (17)	0.0459 (17)	−0.0024 (15)	0.0170 (15)	0.0017 (13)
C56	0.065 (3)	0.0350 (18)	0.0354 (19)	−0.0166 (17)	0.0160 (18)	0.0093 (15)
C57	0.0824 (19)	0.0574 (15)	0.0572 (14)	−0.0113 (13)	0.0265 (14)	0.0118 (12)

### Geometric parameters (Å, °)

**Table:**

Li1—N2	1.9592 (11)	N9—C33	1.3358 (17)
Li1—N2^i^	1.9592 (11)	N9—C34	1.3798 (18)
Li1—N4	1.9650 (11)	N9—C36	1.4906 (17)
Li1—N4^i^	1.9650 (11)	N10—C33	1.3323 (17)
N1—C1	1.3352 (16)	N10—C35	1.3808 (17)
N1—C16^i^	1.3365 (16)	N10—C46	1.4885 (16)
N2—C8	1.3597 (16)	C33—H33	0.9500
N2—C1	1.3625 (16)	C34—C35	1.3481 (19)
N3—C9	1.3411 (16)	C34—H34	0.9500
N3—C8	1.3424 (16)	C35—H35	0.9500
N4—C9	1.3581 (16)	C36—C45	1.529 (2)
N4—C16	1.3617 (16)	C36—C41	1.5307 (18)
C1—C2	1.4603 (17)	C36—C37	1.5309 (19)
C2—C3	1.3895 (18)	C37—C38	1.5337 (19)
C2—C7	1.4020 (18)	C37—H37A	0.9900
C3—C4	1.3859 (19)	C37—H37B	0.9900
C3—H3	0.9500	C38—C42	1.528 (2)
C4—C5	1.396 (2)	C38—C39	1.533 (2)
C4—H4	0.9500	C38—H38	1.0000
C5—C6	1.388 (2)	C39—C40	1.528 (2)
C5—H5	0.9500	C39—H39A	0.9900
C6—C7	1.3914 (18)	C39—H39B	0.9900
C6—H6	0.9500	C40—C44	1.531 (2)
C7—C8	1.4688 (18)	C40—C41	1.5335 (19)
C9—C10	1.4720 (18)	C40—H40	1.0000
C10—C11	1.3923 (18)	C41—H41A	0.9900
C10—C15	1.4016 (18)	C41—H41B	0.9900
C11—C12	1.3892 (19)	C42—C43	1.529 (2)
C11—H11	0.9500	C42—H42A	0.9900
C12—C13	1.399 (2)	C42—H42B	0.9900
C12—H12	0.9500	C43—C44	1.532 (2)
C13—C14	1.3875 (19)	C43—C45	1.535 (2)
C13—H13	0.9500	C43—H43	1.0000
C14—C15	1.3913 (18)	C44—H44A	0.9900
C14—H14	0.9500	C44—H44B	0.9900
C15—C16	1.4593 (18)	C45—H45A	0.9900
C16—N1^i^	1.3365 (16)	C45—H45B	0.9900
Li2—N6	1.9567 (11)	C46—C55	1.5267 (19)
Li2—N6^ii^	1.9567 (11)	C46—C47	1.5287 (18)
Li2—N8	1.9606 (11)	C46—C53	1.5322 (19)
Li2—N8^ii^	1.9606 (11)	C47—C48	1.5347 (19)
N5—C17	1.3343 (17)	C47—H47A	0.9900
N5—C32^ii^	1.3345 (17)	C47—H47B	0.9900
N6—C24	1.3625 (17)	C48—C49	1.526 (2)
N6—C17	1.3656 (17)	C48—C54	1.529 (2)
N7—C24	1.3357 (17)	C48—H48	1.0000
N7—C25	1.3374 (17)	C49—C50	1.534 (2)
N8—C25	1.3667 (17)	C49—H49A	0.9900
N8—C32	1.3672 (17)	C49—H49B	0.9900
C17—C18	1.4634 (18)	C50—C51	1.526 (2)
C18—C19	1.3867 (18)	C50—C55	1.536 (2)
C18—C23	1.3984 (19)	C50—H50	1.0000
C19—C20	1.388 (2)	C51—C52	1.527 (2)
C19—H19	0.9500	C51—H51A	0.9900
C20—C21	1.399 (2)	C51—H51B	0.9900
C20—H20	0.9500	C52—C54	1.530 (2)
C21—C22	1.389 (2)	C52—C53	1.5315 (19)
C21—H21	0.9500	C52—H52	1.0000
C22—C23	1.3910 (19)	C53—H53A	0.9900
C22—H22	0.9500	C53—H53B	0.9900
C23—C24	1.4671 (18)	C54—H54A	0.9900
C25—C26	1.4636 (18)	C54—H54B	0.9900
C26—C27	1.3930 (19)	C55—H55A	0.9900
C26—C31	1.4019 (19)	C55—H55B	0.9900
C27—C28	1.3894 (19)	O1—H1A	0.862 (15)
C27—H27	0.9500	O1—H1B	0.890 (15)
C28—C29	1.400 (2)	O2—C56	1.191 (5)
C28—H28	0.9500	C56—C57^iii^	1.476 (5)
C29—C30	1.388 (2)	C56—C57	1.487 (5)
C29—H29	0.9500	C56—C56^iii^	1.503 (8)
C30—C31	1.3939 (18)	C57—C56^iii^	1.476 (5)
C30—H30	0.9500	C57—H57A	0.948 (18)
C31—C32	1.4628 (18)	C57—H57B	0.931 (18)
C32—N5^ii^	1.3345 (17)	C57—H57C	0.935 (18)
			
N2—Li1—N2^i^	180.00 (8)	N10—C35—H35	126.3
N2—Li1—N4	89.28 (5)	N9—C36—C45	108.13 (11)
N2^i^—Li1—N4	90.72 (5)	N9—C36—C41	109.07 (11)
N2—Li1—N4^i^	90.72 (5)	C45—C36—C41	109.59 (11)
N2^i^—Li1—N4^i^	89.28 (5)	N9—C36—C37	110.33 (10)
N4—Li1—N4^i^	180.00 (8)	C45—C36—C37	109.37 (11)
C1—N1—C16^i^	122.68 (11)	C41—C36—C37	110.32 (11)
C8—N2—C1	107.98 (10)	C36—C37—C38	109.00 (11)
C8—N2—Li1	126.75 (9)	C36—C37—H37A	109.9
C1—N2—Li1	125.25 (8)	C38—C37—H37A	109.9
C9—N3—C8	122.79 (11)	C36—C37—H37B	109.9
C9—N4—C16	107.90 (11)	C38—C37—H37B	109.9
C9—N4—Li1	126.97 (9)	H37A—C37—H37B	108.3
C16—N4—Li1	124.98 (9)	C42—C38—C39	109.88 (12)
N1—C1—N2	128.03 (11)	C42—C38—C37	109.06 (11)
N1—C1—C2	121.71 (11)	C39—C38—C37	109.59 (11)
N2—C1—C2	110.25 (11)	C42—C38—H38	109.4
C3—C2—C7	121.79 (12)	C39—C38—H38	109.4
C3—C2—C1	132.17 (12)	C37—C38—H38	109.4
C7—C2—C1	105.98 (11)	C40—C39—C38	109.39 (11)
C4—C3—C2	117.65 (13)	C40—C39—H39A	109.8
C4—C3—H3	121.2	C38—C39—H39A	109.8
C2—C3—H3	121.2	C40—C39—H39B	109.8
C3—C4—C5	120.76 (13)	C38—C39—H39B	109.8
C3—C4—H4	119.6	H39A—C39—H39B	108.2
C5—C4—H4	119.6	C39—C40—C44	109.89 (12)
C6—C5—C4	121.77 (13)	C39—C40—C41	109.54 (12)
C6—C5—H5	119.1	C44—C40—C41	109.21 (12)
C4—C5—H5	119.1	C39—C40—H40	109.4
C5—C6—C7	117.74 (13)	C44—C40—H40	109.4
C5—C6—H6	121.1	C41—C40—H40	109.4
C7—C6—H6	121.1	C36—C41—C40	109.10 (11)
C6—C7—C2	120.29 (12)	C36—C41—H41A	109.9
C6—C7—C8	133.90 (12)	C40—C41—H41A	109.9
C2—C7—C8	105.72 (11)	C36—C41—H41B	109.9
N3—C8—N2	127.26 (12)	C40—C41—H41B	109.9
N3—C8—C7	122.66 (11)	H41A—C41—H41B	108.3
N2—C8—C7	110.07 (11)	C38—C42—C43	109.65 (12)
N3—C9—N4	126.87 (12)	C38—C42—H42A	109.7
N3—C9—C10	122.93 (11)	C43—C42—H42A	109.7
N4—C9—C10	110.20 (11)	C38—C42—H42B	109.7
C11—C10—C15	120.37 (12)	C43—C42—H42B	109.7
C11—C10—C9	134.17 (12)	H42A—C42—H42B	108.2
C15—C10—C9	105.46 (11)	C42—C43—C44	109.72 (12)
C12—C11—C10	117.89 (12)	C42—C43—C45	109.12 (11)
C12—C11—H11	121.1	C44—C43—C45	109.47 (12)
C10—C11—H11	121.1	C42—C43—H43	109.5
C11—C12—C13	121.46 (12)	C44—C43—H43	109.5
C11—C12—H12	119.3	C45—C43—H43	109.5
C13—C12—H12	119.3	C40—C44—C43	109.39 (12)
C14—C13—C12	120.97 (13)	C40—C44—H44A	109.8
C14—C13—H13	119.5	C43—C44—H44A	109.8
C12—C13—H13	119.5	C40—C44—H44B	109.8
C13—C14—C15	117.54 (13)	C43—C44—H44B	109.8
C13—C14—H14	121.2	H44A—C44—H44B	108.2
C15—C14—H14	121.2	C36—C45—C43	109.16 (11)
C14—C15—C10	121.77 (12)	C36—C45—H45A	109.8
C14—C15—C16	132.13 (12)	C43—C45—H45A	109.8
C10—C15—C16	106.10 (11)	C36—C45—H45B	109.8
N1^i^—C16—N4	128.09 (12)	C43—C45—H45B	109.8
N1^i^—C16—C15	121.59 (12)	H45A—C45—H45B	108.3
N4—C16—C15	110.31 (11)	N10—C46—C55	109.00 (11)
N6—Li2—N6^ii^	180.00 (7)	N10—C46—C47	109.81 (10)
N6—Li2—N8	90.47 (5)	C55—C46—C47	110.10 (11)
N6^ii^—Li2—N8	89.53 (5)	N10—C46—C53	108.16 (10)
N6—Li2—N8^ii^	89.53 (5)	C55—C46—C53	110.19 (11)
N6^ii^—Li2—N8^ii^	90.47 (5)	C47—C46—C53	109.55 (11)
N8—Li2—N8^ii^	180.00 (3)	C46—C47—C48	108.73 (11)
C17—N5—C32^ii^	122.18 (11)	C46—C47—H47A	109.9
C24—N6—C17	107.97 (11)	C48—C47—H47A	109.9
C24—N6—Li2	125.63 (9)	C46—C47—H47B	109.9
C17—N6—Li2	126.35 (9)	C48—C47—H47B	109.9
C24—N7—C25	122.74 (12)	H47A—C47—H47B	108.3
C25—N8—C32	107.97 (11)	C49—C48—C54	109.21 (12)
C25—N8—Li2	125.43 (9)	C49—C48—C47	109.43 (12)
C32—N8—Li2	126.19 (9)	C54—C48—C47	109.83 (11)
N5—C17—N6	127.84 (12)	C49—C48—H48	109.5
N5—C17—C18	122.04 (12)	C54—C48—H48	109.5
N6—C17—C18	110.13 (11)	C47—C48—H48	109.5
C19—C18—C23	121.39 (12)	C48—C49—C50	109.44 (12)
C19—C18—C17	132.72 (13)	C48—C49—H49A	109.8
C23—C18—C17	105.89 (11)	C50—C49—H49A	109.8
C18—C19—C20	117.78 (13)	C48—C49—H49B	109.8
C18—C19—H19	121.1	C50—C49—H49B	109.8
C20—C19—H19	121.1	H49A—C49—H49B	108.2
C19—C20—C21	120.99 (13)	C51—C50—C49	110.30 (13)
C19—C20—H20	119.5	C51—C50—C55	109.30 (12)
C21—C20—H20	119.5	C49—C50—C55	108.73 (13)
C22—C21—C20	121.20 (13)	C51—C50—H50	109.5
C22—C21—H21	119.4	C49—C50—H50	109.5
C20—C21—H21	119.4	C55—C50—H50	109.5
C21—C22—C23	117.79 (13)	C50—C51—C52	109.91 (12)
C21—C22—H22	121.1	C50—C51—H51A	109.7
C23—C22—H22	121.1	C52—C51—H51A	109.7
C22—C23—C18	120.83 (12)	C50—C51—H51B	109.7
C22—C23—C24	133.03 (13)	C52—C51—H51B	109.7
C18—C23—C24	106.13 (11)	H51A—C51—H51B	108.2
N7—C24—N6	127.88 (12)	C51—C52—C54	108.96 (12)
N7—C24—C23	122.24 (12)	C51—C52—C53	109.23 (12)
N6—C24—C23	109.88 (11)	C54—C52—C53	109.53 (11)
N7—C25—N8	127.62 (12)	C51—C52—H52	109.7
N7—C25—C26	122.45 (12)	C54—C52—H52	109.7
N8—C25—C26	109.93 (11)	C53—C52—H52	109.7
C27—C26—C31	120.84 (12)	C52—C53—C46	109.16 (11)
C27—C26—C25	132.99 (12)	C52—C53—H53A	109.8
C31—C26—C25	106.09 (11)	C46—C53—H53A	109.8
C28—C27—C26	117.44 (13)	C52—C53—H53B	109.8
C28—C27—H27	121.3	C46—C53—H53B	109.8
C26—C27—H27	121.3	H53A—C53—H53B	108.3
C27—C28—C29	121.79 (13)	C48—C54—C52	110.00 (12)
C27—C28—H28	119.1	C48—C54—H54A	109.7
C29—C28—H28	119.1	C52—C54—H54A	109.7
C30—C29—C28	120.83 (13)	C48—C54—H54B	109.7
C30—C29—H29	119.6	C52—C54—H54B	109.7
C28—C29—H29	119.6	H54A—C54—H54B	108.2
C29—C30—C31	117.62 (13)	C46—C55—C50	108.86 (12)
C29—C30—H30	121.2	C46—C55—H55A	109.9
C31—C30—H30	121.2	C50—C55—H55A	109.9
C30—C31—C26	121.48 (12)	C46—C55—H55B	109.9
C30—C31—C32	132.41 (13)	C50—C55—H55B	109.9
C26—C31—C32	106.03 (11)	H55A—C55—H55B	108.3
N5^ii^—C32—N8	127.75 (12)	H1A—O1—H1B	105.0 (18)
N5^ii^—C32—C31	122.23 (12)	O2—C56—C57^iii^	120.8 (4)
N8—C32—C31	109.99 (11)	O2—C56—C57	120.0 (4)
C33—N9—C34	108.36 (11)	C57^iii^—C56—C57	119.1 (3)
C33—N9—C36	124.24 (11)	O2—C56—C56^iii^	177.4 (5)
C34—N9—C36	127.12 (11)	C57^iii^—C56—C56^iii^	59.9 (3)
C33—N10—C35	108.33 (11)	C57—C56—C56^iii^	59.2 (3)
C33—N10—C46	127.08 (11)	C56^iii^—C57—C56	60.9 (3)
C35—N10—C46	124.55 (11)	C56^iii^—C57—H57A	156.5 (19)
N10—C33—N9	108.73 (12)	C56—C57—H57A	112.2 (15)
N10—C33—H33	125.6	C56^iii^—C57—H57B	76.6 (18)
N9—C33—H33	125.6	C56—C57—H57B	116.3 (15)
C35—C34—N9	107.22 (12)	H57A—C57—H57B	88 (2)
C35—C34—H34	126.4	C56^iii^—C57—H57C	101.4 (18)
N9—C34—H34	126.4	C56—C57—H57C	115.1 (15)
C34—C35—N10	107.36 (12)	H57A—C57—H57C	102 (3)
C34—C35—H35	126.3	H57B—C57—H57C	118 (3)
			
N4—Li1—N2—C8	−0.79 (11)	C24—N7—C25—N8	−3.2 (2)
N4^i^—Li1—N2—C8	179.21 (11)	C24—N7—C25—C26	176.20 (12)
N4—Li1—N2—C1	−178.85 (10)	C32—N8—C25—N7	179.25 (13)
N4^i^—Li1—N2—C1	1.15 (10)	Li2—N8—C25—N7	6.3 (2)
N2—Li1—N4—C9	1.91 (11)	C32—N8—C25—C26	−0.18 (14)
N2^i^—Li1—N4—C9	−178.09 (11)	Li2—N8—C25—C26	−173.14 (8)
N2—Li1—N4—C16	176.80 (11)	N7—C25—C26—C27	−3.0 (2)
N2^i^—Li1—N4—C16	−3.20 (11)	N8—C25—C26—C27	176.45 (14)
C16^i^—N1—C1—N2	2.2 (2)	N7—C25—C26—C31	−179.63 (12)
C16^i^—N1—C1—C2	−179.29 (12)	N8—C25—C26—C31	−0.17 (14)
C8—N2—C1—N1	177.65 (12)	C31—C26—C27—C28	0.03 (19)
Li1—N2—C1—N1	−3.99 (19)	C25—C26—C27—C28	−176.19 (13)
C8—N2—C1—C2	−1.00 (14)	C26—C27—C28—C29	−0.5 (2)
Li1—N2—C1—C2	177.36 (8)	C27—C28—C29—C30	0.8 (2)
N1—C1—C2—C3	5.0 (2)	C28—C29—C30—C31	−0.5 (2)
N2—C1—C2—C3	−176.21 (13)	C29—C30—C31—C26	0.0 (2)
N1—C1—C2—C7	−177.94 (12)	C29—C30—C31—C32	176.01 (14)
N2—C1—C2—C7	0.80 (14)	C27—C26—C31—C30	0.24 (19)
C7—C2—C3—C4	−0.38 (19)	C25—C26—C31—C30	177.36 (12)
C1—C2—C3—C4	176.23 (13)	C27—C26—C31—C32	−176.71 (12)
C2—C3—C4—C5	0.2 (2)	C25—C26—C31—C32	0.41 (13)
C3—C4—C5—C6	0.3 (2)	C25—N8—C32—N5^ii^	−177.29 (13)
C4—C5—C6—C7	−0.5 (2)	Li2—N8—C32—N5^ii^	−4.40 (19)
C5—C6—C7—C2	0.3 (2)	C25—N8—C32—C31	0.45 (14)
C5—C6—C7—C8	−175.63 (14)	Li2—N8—C32—C31	173.34 (8)
C3—C2—C7—C6	0.2 (2)	C30—C31—C32—N5^ii^	0.9 (2)
C1—C2—C7—C6	−177.23 (12)	C26—C31—C32—N5^ii^	177.34 (12)
C3—C2—C7—C8	177.10 (12)	C30—C31—C32—N8	−177.02 (14)
C1—C2—C7—C8	−0.29 (13)	C26—C31—C32—N8	−0.55 (14)
C9—N3—C8—N2	−2.3 (2)	C35—N10—C33—N9	−0.38 (16)
C9—N3—C8—C7	176.25 (12)	C46—N10—C33—N9	177.78 (12)
C1—N2—C8—N3	179.48 (12)	C34—N9—C33—N10	0.13 (16)
Li1—N2—C8—N3	1.15 (19)	C36—N9—C33—N10	−174.13 (12)
C1—N2—C8—C7	0.81 (14)	C33—N9—C34—C35	0.17 (17)
Li1—N2—C8—C7	−177.52 (8)	C36—N9—C34—C35	174.22 (13)
C6—C7—C8—N3	−2.7 (2)	N9—C34—C35—N10	−0.40 (17)
C2—C7—C8—N3	−179.05 (12)	C33—N10—C35—C34	0.48 (17)
C6—C7—C8—N2	176.03 (14)	C46—N10—C35—C34	−177.73 (13)
C2—C7—C8—N2	−0.30 (14)	C33—N9—C36—C45	64.57 (16)
C8—N3—C9—N4	3.6 (2)	C34—N9—C36—C45	−108.59 (15)
C8—N3—C9—C10	−175.40 (12)	C33—N9—C36—C41	−176.32 (13)
C16—N4—C9—N3	−179.22 (12)	C34—N9—C36—C41	10.52 (19)
Li1—N4—C9—N3	−3.61 (19)	C33—N9—C36—C37	−55.00 (17)
C16—N4—C9—C10	−0.14 (14)	C34—N9—C36—C37	131.84 (14)
Li1—N4—C9—C10	175.46 (8)	N9—C36—C37—C38	179.77 (11)
N3—C9—C10—C11	−1.2 (2)	C45—C36—C37—C38	60.96 (14)
N4—C9—C10—C11	179.65 (14)	C41—C36—C37—C38	−59.65 (14)
N3—C9—C10—C15	178.30 (12)	C36—C37—C38—C42	−60.66 (14)
N4—C9—C10—C15	−0.82 (14)	C36—C37—C38—C39	59.67 (14)
C15—C10—C11—C12	−0.87 (19)	C42—C38—C39—C40	59.37 (15)
C9—C10—C11—C12	178.61 (13)	C37—C38—C39—C40	−60.45 (15)
C10—C11—C12—C13	0.1 (2)	C38—C39—C40—C44	−59.55 (15)
C11—C12—C13—C14	0.4 (2)	C38—C39—C40—C41	60.44 (15)
C12—C13—C14—C15	−0.2 (2)	N9—C36—C41—C40	−178.95 (11)
C13—C14—C15—C10	−0.6 (2)	C45—C36—C41—C40	−60.75 (15)
C13—C14—C15—C16	179.63 (13)	C37—C36—C41—C40	59.72 (15)
C11—C10—C15—C14	1.1 (2)	C39—C40—C41—C36	−59.79 (15)
C9—C10—C15—C14	−178.49 (12)	C44—C40—C41—C36	60.61 (15)
C11—C10—C15—C16	−179.02 (12)	C39—C38—C42—C43	−59.47 (15)
C9—C10—C15—C16	1.36 (13)	C37—C38—C42—C43	60.67 (14)
C9—N4—C16—N1^i^	−178.37 (13)	C38—C42—C43—C44	59.57 (15)
Li1—N4—C16—N1^i^	5.92 (19)	C38—C42—C43—C45	−60.37 (15)
C9—N4—C16—C15	1.03 (14)	C39—C40—C44—C43	59.74 (15)
Li1—N4—C16—C15	−174.68 (8)	C41—C40—C44—C43	−60.45 (16)
C14—C15—C16—N1^i^	−2.3 (2)	C42—C43—C44—C40	−59.58 (16)
C10—C15—C16—N1^i^	177.90 (12)	C45—C43—C44—C40	60.15 (16)
C14—C15—C16—N4	178.29 (14)	N9—C36—C45—C43	179.11 (11)
C10—C15—C16—N4	−1.54 (14)	C41—C36—C45—C43	60.33 (15)
N8—Li2—N6—C24	0.27 (11)	C37—C36—C45—C43	−60.72 (14)
N8^ii^—Li2—N6—C24	−179.73 (11)	C42—C43—C45—C36	60.18 (15)
N8—Li2—N6—C17	−176.88 (11)	C44—C43—C45—C36	−59.92 (15)
N8^ii^—Li2—N6—C17	3.12 (11)	C33—N10—C46—C55	120.09 (15)
N6—Li2—N8—C25	−4.10 (11)	C35—N10—C46—C55	−62.03 (17)
N6^ii^—Li2—N8—C25	175.90 (11)	C33—N10—C46—C47	−0.60 (19)
N6—Li2—N8—C32	−175.80 (11)	C35—N10—C46—C47	177.28 (13)
N6^ii^—Li2—N8—C32	4.20 (11)	C33—N10—C46—C53	−120.10 (14)
C32^ii^—N5—C17—N6	0.5 (2)	C35—N10—C46—C53	57.77 (17)
C32^ii^—N5—C17—C18	−179.08 (12)	N10—C46—C47—C48	−179.67 (11)
C24—N6—C17—N5	−179.52 (13)	C55—C46—C47—C48	60.31 (15)
Li2—N6—C17—N5	−2.0 (2)	C53—C46—C47—C48	−61.02 (14)
C24—N6—C17—C18	0.07 (15)	C46—C47—C48—C49	−59.97 (15)
Li2—N6—C17—C18	177.63 (9)	C46—C47—C48—C54	59.90 (15)
N5—C17—C18—C19	0.0 (2)	C54—C48—C49—C50	−59.32 (16)
N6—C17—C18—C19	−179.67 (14)	C47—C48—C49—C50	60.93 (16)
N5—C17—C18—C23	179.73 (12)	C48—C49—C50—C51	58.81 (16)
N6—C17—C18—C23	0.11 (15)	C48—C49—C50—C55	−61.02 (16)
C23—C18—C19—C20	−0.2 (2)	C49—C50—C51—C52	−58.90 (15)
C17—C18—C19—C20	179.54 (14)	C55—C50—C51—C52	60.58 (15)
C18—C19—C20—C21	−0.3 (2)	C50—C51—C52—C54	59.22 (15)
C19—C20—C21—C22	0.6 (2)	C50—C51—C52—C53	−60.39 (15)
C20—C21—C22—C23	−0.4 (2)	C51—C52—C53—C46	59.54 (15)
C21—C22—C23—C18	−0.1 (2)	C54—C52—C53—C46	−59.72 (15)
C21—C22—C23—C24	−179.03 (14)	N10—C46—C53—C52	−179.12 (11)
C19—C18—C23—C22	0.4 (2)	C55—C46—C53—C52	−60.06 (14)
C17—C18—C23—C22	−179.42 (12)	C47—C46—C53—C52	61.21 (14)
C19—C18—C23—C24	179.58 (12)	C49—C48—C54—C52	60.81 (15)
C17—C18—C23—C24	−0.23 (14)	C47—C48—C54—C52	−59.20 (15)
C25—N7—C24—N6	−1.5 (2)	C51—C52—C54—C48	−60.47 (15)
C25—N7—C24—C23	178.69 (12)	C53—C52—C54—C48	58.95 (16)
C17—N6—C24—N7	179.97 (13)	N10—C46—C55—C50	178.57 (11)
Li2—N6—C24—N7	2.4 (2)	C47—C46—C55—C50	−60.91 (15)
C17—N6—C24—C23	−0.22 (15)	C53—C46—C55—C50	60.03 (15)
Li2—N6—C24—C23	−177.80 (9)	C51—C50—C55—C46	−59.87 (16)
C22—C23—C24—N7	−0.8 (2)	C49—C50—C55—C46	60.58 (16)
C18—C23—C24—N7	−179.89 (12)	O2—C56—C57—C56^iii^	177.2 (5)
C22—C23—C24—N6	179.34 (14)	C57^iii^—C56—C57—C56^iii^	0.000 (1)
C18—C23—C24—N6	0.28 (15)		

Symmetry codes: (i) −*x*+2, −*y*, −*z*; (ii) −*x*, −*y*+1, −*z*; (iii) −*x*, −*y*+1, −*z*+1.

### Hydrogen-bond geometry (Å, °)

**Table:**

*D*—H···*A*	*D*—H	H···*A*	*D*···*A*	*D*—H···*A*
O1—H1A···N6	0.86 (2)	2.40 (2)	3.1618 (17)	147 (2)
O1—H1B···N3^iv^	0.89 (2)	2.03 (2)	2.8911 (17)	164 (2)
C33—H33···O1	0.95	2.18	3.127 (2)	171
C37—H37A···O1	0.99	2.52	3.493 (2)	166

Symmetry codes: (iv) −*x*+1, −*y*+1, −*z*.
